# Dorsal hand coverage

**DOI:** 10.1186/1753-6561-9-S3-A59

**Published:** 2015-05-19

**Authors:** Roberto Adani

**Affiliations:** 1 Department of Hand Surgery, Azienda Ospedaliera Universitaria Integrata Verona, Verona, 37134, Italy

## 

The dorsum of the hand is a very specialized region with thin and fragile skin characterized by poor subcutaneous tissue. The dorsal aspect of the hand is frequently prone to different types of injuries (crush, degloving, hot press, friction…) resulting in exposed tendons and bone. The treatment of cutaneous defects may be obtained with local pedicle flaps, distant pedicle flaps or with free flaps. The choice of which technique to employ depends mainly on the size of the defect. For medium-sized defects (less than 20 cm^2^) pedicle flaps represent a simple solution; they offer pliable skin that is very similar to the skin of the dorsum of the hand.

The options for coverage with a regional pedicle flap include the radial forearm flap [1-3], the ulnar forearm flap [4,5], the posterior interosseous flap [6-9] and the more recent distal ulnar and radial artery perforator-based flaps [10-13].

The radial forearm flap is suitable to cover the dorsal aspect of the hand; its use has been criticized because it requires the sacrifice of a major artery in an already traumatized hand and the appearance of the donor site result is not always satisfactory. However if it is employed to cover medium-sized defects with direct closure of the donor site and without jeopardizing the vascularisation of the hand, this procedure may still have a role for hand reconstruction. The ulnar pedicle flap and the posterior interosseous flap have not gained the same degree of consensus. This is probably due to the concern of harvesting the more dominant ulnar artery with the ulnar forearm flap. The posterior interosseous pedicle island flap has some advantages over the radial forearm flap: it is thinner, there is less morbidity at the donor site, and the major artery is preserved. Its greatest drawback is the limited sizes available for the flap: closure of the donor site can be achieved only if it is less than 3 to 4 cm wide, otherwise poor cosmetic results can be observed when the donor area is skin grafted.

We believe that these pedicle flaps are still useful [14] in the armamentarium of hand surgeons, particularly of young surgeons who are frequently on call during the night. In our practice the posterior interosseous flap and the radial forearm flap are used only when the donor site is closed directly without the need for skin graft.

The perforator flaps harvested from the forearm (the radial artery perforator flap or the ulnar artery perforator flap) are used as either fasciocutaneous flaps or adipofascial flaps, in this latter case in combination with a full-thickness skin graft. These flaps show some disadvantages: they can be used only to cover moderate-sized defects; their pivot point is located more proximally than in traditional pedicle flaps therefore it is difficult to extend them to cover the distal dorsal aspect of the hand completely; finally, the adipofascial pedicle is relatively bulky after rotation making direct closure dangerous for the pedicle. Moreover they should be considered very carefully in patients with complex hand injuries to the forearm with possible damage to the perforators of the radial or ulnar arteries [15].

The use of pedicle flaps in cases characterized by large skin defects (more than 20 cm2) is more restricted. The donor skin defect after raising a pedicle radial forearm flap has been criticized because of the poor donor-site result. These problems can be partially solved by using the retrograde radial forearm fascia-fat flap, which removes only the fascia and fat layers of the forearm tissue and leaves the forearm skin intact [16,17]. Another possibility is to divide the radial forearm flap into different sections following the perforators by using a long narrow flap. In this way it is possible to perform a primary closure of the donor site [18].

Fasciocutaneous flaps are the most commonly employed flaps to manage large defects of the dorsal hand [19-21]. The choice of which flap to use depends on many factors; the cosmetic match of the skin surrounding the defect, the reduction of donor site morbidity and the simultaneous approach to wound debridement and flap harvesting [19]. The anterolateral thigh flap has recently received attention [20,22-24] from hand surgeons and now represents a good alternative to other fasciocutaneous flaps. The lateral arm flap [19,20,25-27] is sometimes limited by its short pedicle and the scapular flap [20] requires a change of position during the operation, hindering a two-team approach. Compared to these flaps, the anterolateral thigh flap has numerous advantages: simultaneous flap elevation and preparation; shorter operative time; longer vascular pedicle (approximately 10 cm long); a large skin paddle can be harvested even when only a single perforator is available [23]. The flap can be thinned to approximately 3 to 4 mm by removing a considerable amount of fatty tissue before it is transferred on the hand. This procedure is important to obtain an optimal match between the donor tissue and the area to be reconstructed with the flap.

A useful technique is also represented by the groin flap [19,26] which can be used as a pedicle or a free flap. The free groin flap presents some advantages: it does not require immobilization of the hand; it allows the use of a large flap; it can be harvested very thin [28].

Instead of fasciocutaneous flaps, some authors [29,30] suggest the use of muscle flaps covered with a split-thickness skin graft. Muscle flaps are tailored to the size of the defect; the most commonly employed are the partial superior latissimus or the partial medial rectus flaps. With this procedure the debulking seems unnecessary achieving a good cosmetic result in the hand and leaving the majority of the donor muscle intact. Our experience with muscle flaps is still reserved to those cases characterized by defects with a dead space or cases of resolved severe infection.
